# Relevance of Next-Generation Sequencing in the Diagnosis of Thalassemia and Hemoglobinopathies: The Experience of Four Italian Diagnostic Hubs

**DOI:** 10.3390/genes16010028

**Published:** 2024-12-27

**Authors:** Rita Selvatici, Valentina Guida, Massimo Maffei, Milena Agata Irrera, Alice Margutti, Paola Bisceglia, Massimo Mogni, Erica Melchionda, Giuseppina Stoico, Nicoletta Grifone, Laura Bocciardo, Simone Salerio, Vittoria Nagliati, Angela Alberico, Giusy Tringali, Cristina Melles, Alessandro De Luca, Alessandra Ferlini, Domenico Coviello, Cristina Curcio

**Affiliations:** 1 Unit of Medical Genetics, Department of Medical Sciences, University Hospital S. Anna Ferrara, 44121 Ferrara, Italy; rita.selvatici@unife.it (R.S.); alice.margutti@unife.it (A.M.); giuseppina.stoico@ospfe.it (G.S.); vittoria.nagliati@unife.it (V.N.); fla@unife.it (A.F.); 2 Unit of Medical Genetics, Department of Mother and Child, University Hospital S. Anna Ferrara, 44121 Ferrara, Italy; 3 Division of Medical Genetics, Fondazione IRCCS-Casa Sollievo della Sofferenza, 71013 San Giovanni Rotondo, Italy; v.guida@operapadrepio.it (V.G.); p.bisceglia@operapadrepio.it (P.B.); n.grifone@operapadrepio.it (N.G.); a.alberico@operapadrepio.it (A.A.); a.deluca@operapadrepio.it (A.D.L.); 4 Laboratory of Human Genetics, IRCCS Istituto Giannina Gaslini, 16147 Genoa, Italy; massimomaffei@gaslini.org (M.M.); massimomogni@gaslini.org (M.M.); laurabocciardo@gaslini.org (L.B.); giusytringali@gaslini.org (G.T.); 5 Laboratory of Medical Genetics, Clinical Pathology UOC, Fondazione IRCCS Ca’ Granda Ospedale Maggiore Policlinico, 20122 Milan, Italy; milena.irrera@policlinico.mi.it (M.A.I.); erica.melchionda@policlinico.mi.it (E.M.); simone.salerio@policlinico.mi.it (S.S.); cristina.melles@policlinico.mi.it (C.M.); cristina.curcio@policlinico.mi.it (C.C.)

**Keywords:** thalassemias, hemoglobinopathies, next-generation sequencing approach

## Abstract

Thalassemias and hemoglobinopathies are among the most common genetic diseases worldwide and have a significant impact on public health. The decreasing cost of next-generation sequencing (NGS) has quickly enabled the development of new assays that allow for the simultaneous analysis of small nucleotide variants (SNVs) and copy number variants (CNVs) as deletions/duplications of α- and β-globin genes. Background/Objectives: This study highlighted the efficacy and rapid identification of all types of mutations in the α- and β-globin genes, including silent variants, using the Devyser Thalassemia NGS kit. Furthermore, we report the frequency of mutations identified in a total population of 2649 individuals recruited from four Italian Medical Genetics Laboratories. Methods: All samples were first hematologically characterized, and sequence analysis was conducted by using the Devyser Thalassemia NGS kit. All variants were also validated in an independent sample by a conventional molecular test. Results: A total of 1789 subjects were identified with genetic variants in the globin genes, of which 966 (53.9%) had variations in the β-gene, 480 (26.8%) had variations in the α-gene; and 307 (17.1%) had variations in both α- and β-genes. Variant analysis evidenced a heterogeneous mutation spectrum enriched with variants not usually observed in the Italian population. Conclusions: This study showed the high effectiveness and the rapid identification of all mutation types in both α- and β-globin genes, including silent variants. It should be emphasized that the NGS approach greatly shortens turnaround reporting times, overcoming the classic diagnostic flowchart which envisages multistep, subsequent, diagnostic approaches, often requiring long resolution times.

## 1. Introduction

Thalassemias and hemoglobinopathies are a group of inherited blood autosomal recessive disorders characterized by abnormalities in the production of hemoglobin. These disorders are among the most common genetic diseases worldwide and have a significant impact on public health. It is estimated that 1.5% to 5% of the world’s population carries a gene mutation for either α-thalassemia or β-thalassemia. The prevalence of specific types of thalassemias and hemoglobinopathies varies among populations due to the selective advantage conferred by carrying a single mutation, such as genetic advantage against malaria, genetic diversity within populations and historical migration patterns [1]. α-thalassemias are more common in Southeast Asia, with more than 30% of carrier frequency, while β-thalassemias are more prevalent in the Mediterranean regions, India, Thailand, and Cambogia. In Italy alone, there are an estimated 7000 thalassemia patients and around 3 million individuals who carry the mutant α- or β-thalassemia gene. These carriers exhibit a wide range of phenotypes, which depend on the specific genetic mutations and their effects on hemoglobin production and function [2]. Mutations in globin gene clusters can cause a range of health problems, including chronic anemia, jaundice, splenomegaly (enlarged spleen), growth retardation, and skeletal abnormalities [3]. Severe forms of these disorders can be life-threatening if left untreated. Often lifelong medical care, including regular blood transfusions [4], red blood cell exchange, iron chelation therapy to manage iron overload, and sometimes bone marrow transplants are required, significantly impacting on the quality of life of these patients [5]. Conventional molecular techniques provide accurate and reliable results but are labor-intensive, time-consuming, and often limited in their ability to analyze large genomic regions or detect complex genetic alterations like copy number variants (CNVs) [6]. The evolution of molecular techniques in the field of genetic diagnosis of thalassemias and hemoglobinopathies has been marked by the introduction of next generation sequencing (NGS), enabling comprehensive analysis of the α- and β-globin gene clusters in a single assay and facilitating the detection of single nucleotide variants (SNVs), insertions/deletions (indels), and copy number variants (CNVs) associated with thalassemias and hemoglobinopathies [7]. NGS can: (i) identify carriers, enabling the identification of individuals at risk of passing the mutation to their offspring; (ii) when applied to prenatal testing, allow early variants detection and informed reproductive decision-making; (iii) identify individuals presenting with clinical symptoms of the disease, facilitating appropriate management and treatment. The aim of this study was to discuss the different aspects of the application of NGS in the diagnosis of thalassemias and hemoglobinopathies, describing its limitations and advantages, exploring the genetic landscape of these conditions across different population groups, as well as the resolution of complex hematological conditions for which common molecular techniques present several inadequacies. Furthermore, the NGS approach offers the advantage of identifying silent variants, which are often overlooked by conventional diagnostic methods. Although these variants may not have an immediate phenotypic impact, showing no significant clinical symptoms or significant changes in the hemoglobin profile, their inclusion in the analysis provides a comprehensive understanding of the individual’s genetic profile and contributes to a more accurate assessment of carrier status determination, disease progression potential, couple risks, and family planning.

## 2. Materials and Methods

### 2.1. Patient Enrollment

In this study, a total of 2649 patients (1180 males; 1469 females) were recruited from four Italian Medical Genetics Laboratories, for the characterization of hemoglobinopathy: 749 patients (326 males; 423 females) from IRCCS Ca’ Granda Foundation Policlinico Maggiore Hospital in Milan (sent both from the internal departments of the Foundation and from external or extra-regional affiliated Clinical Centres); 846 patients (356 male; 490 female) from IRCCS Istituto Giannina Gaslini in Genoa; 892 patients (424 male; 468 female) from U.O.L. of Medical Genetics Unit Dept. Medical Sciences & Dept. of Reproduction and Growth, University-Hospital S.Anna, Ferrara; 162 patients (74 male; 88 female) from Fondazione Ospedale Casa Sollievo della Sofferenza Istituto Mendel, Rome. The NGS tests were performed on all patients after obtaining the informed consent for specific analysis. Genetic variants in the globin genes were presented in 1789 patients and 860 patients were negative.

### 2.2. Primary Hematological Screening

All peripheral blood samples from patients were screened with complete routine blood examination: full blood count, study of the morphology of the red blood cells, evaluation of iron status, and Hb analysis were performed according to local standard protocols by the respective laboratories. Cut-off values indicating suspected thalassemia carriers included the following: (1) MCV (mean corpuscular volume) < 80/82 fl and/or MHC (mean corpuscular hemoglobin) < 27 pg; (2) low or normal HbA2 concentration ≤ 2.0–3.1% (suspected α-thalassemia); (3) for HbA2 concentration > 3.5% (suspected β-thalassemia). The definitive diagnosis of these pathologies can only be determined by molecular analysis.

### 2.3. Genomic DNA Extraction

Genomic DNA was extracted automatically using the QIASIMPHONY instrument (QIAGEN Group) from periphery whole blood using the QIAamp DNA Blood Mini e Midi Kit (Qiagen, Hilden, Germany) or from 200 µL of peripheral blood drawn in EDTA using the manual NucleoSpin Blood kit (Macherey-Nagel, Dueren, Germany). The DNA samples quantification and quantity control were measured with QIAxpert, a high-speed microfluidic UV/VIS spectrophotometer (Qiagen, Hilden, Germany), or a Nanodrop ND-1000 spectrophotometer (NanoDrop Technologies, Inc, Wilmington, DE, USA). A Qubit 3 Fluorometer (ThermoFisher Scientific, Waltham, MA, USA) was used for all NGS library dilutions and assays.

### 2.4. NGS Sequencing

Targeted NGS for thalassemia libraries was obtained using the Devyser Thalassemia NGS assay kit CE-IVD 8-A106 (Devyser, Hägersten, Sweden) following the manufacturer’s instructions [8]. Briefly, 10 ng (2 ng/μL) of genomic DNA was used to amplify the HBA1, HBA2, and HBB genes in a single-tube multiplex reaction; this PCR-based library was diluted and used to incorporate molecular barcodes and adapter sequences into each amplicon by a second PCR reaction. Amplicon libraries were pooled to generate a sequencing library that was purified using the Devyser Library Clean kit (Devyser AB) and quantified using the High Sensitivity Qubit quantification kit (Life Technologies, Carlsbad, CA, USA). The final library was normalized to a concentration of 2 nM and sequenced using Illumina MiSeq platform (Illumina, San Diego, CA, USA) according to the manufacturer’s instructions to generate pair-end reads. A specific bioinformatic software (Devyser Amplicon Suit v3.7) workflow was used for data analysis. This targeted NGS assay specifically identified sequence variants in HBA1, HBA2, and HBB and common sequence variants, including exon spanning copy number variations (CNVs), small or large insertion and deletions (indels), and single-nucleotide variants (SNVs).

All the variants identified by NGS analysis were also validated in an independent sample by a conventional molecular test (Sanger sequencing, reverse dot blot, GAP-PCR or MLPA assay). Validation was omitted only in the presence of a previously run high-performance liquid chromatography (HPLC) analysis that highlighted specific hemoglobin subtypes such as HbS, HbC, HbE, HbD, and Hb-Lepore.

### 2.5. Sanger Sequencing

Sanger sequencing of the α-, β-, δ-globin genes and γ-promoters were performed according to local standard protocols by the respective laboratories using dedicated primers [9,10,11]. The sequencing was performed using the BigDye Terminator v3.1 Cycle Sequencing Kit (Applied Biosystems; Thermo Fisher Scientific, Inc.) and the ABI 3130xl, ABI 3500 DX and 3730 Automated Sequencers (Applied Biosystems, Foster City, CA, USA). The sequencing results were interpreted using sequencing analysis software (SeqScape v2.6; Sequencing Analysis 5.3.1) updated to the last available version at the time of sequencing. The hemoglobin variants were named as reported in HbVar [12] following the classic nomenclature and as indicated by HGVS recommendations [13].

### 2.6. Reverse Dot Blot

Reverse dot blot (RDB) analysis [14] for β-globin gene was performed using two commercial kits AC091 and AC104 (Nuclear Laser Medicine, NLM) [15] allowing the detection of 25 and 14 common β-globin variants in the Italian population including Hb Lepore-Boston and Delta β Sicilian deletions and anti-3.7 α-globin gene duplication.

The IRCCS Ca’ Granda Foundation Policlinico Maggiore Hospital (Milan) performed reverse dot blot using a commercial kit for the detection of 23 common β-thalassemia mutations in the Italian population (ViennaLab—β-Globin StripAssay MED) [16].

For analysis of the α-globin genes, the commercial α-globin test kit AC099 (Nuclear Laser Medicine, NLM) [15] was used, which allows the detection of 22 alterations including 7 deletions common in α-thalassemic populations and 15 point mutations frequent in the Italian population.

### 2.7. Multiplex Ligation-Dependent Probe Amplification

Multiplex ligation-dependent probe amplification (MLPA) analysis, was performed on genomic DNA by Probemix P140 HBA [17] and MLPA probemix P102 HBB kit [18] (MRC-Holland) to confirm deletion/duplication analysis of the α- and β-globin locus, respectively.

The analysis recognizes both gains and deletions of the entire HBB, HBD, HBG1, HBG2, HBA1, HBA2 genes and microdeletions of one or more intron/exons.

All reactions (denaturation, ligation, and PCR) were performed following the manufacturer’s instructions. PCR products were run on a 3130xl automated sequencer (Applied Biosystems, Foster City, CA, USA) with ROX500 size standard and data were analyzed using Coffalyser v.140721.1958 software (Applied Biosystems, Foster City). In selected cases, RDB analysis was carried out to define the possible co-inheritance of simultaneous gains/deletions.

### 2.8. GAP-PCR

Common deletions/duplication (such as α-3.7, -4,2, -MED, anti-3,7 and anti-4.2) in the α-globin locus were detected using GAP_PCR with an initial heat activation step of 15 min at 95 °C followed by 35 cycles of 95 °C for 1 min, 65 °C for 1 min, and 72 °C for 2 min 30 s, and then an extension step of 72 °C for 10 min, as described by Liu et al. [19].

## 3. Results

A total of 2649 subjects were enrolled in this study, including 1180 males and 1469 females. NGS sequencing was performed on all patients after signing the informed consent, accompanied by the diagnostic level I test: blood count, evaluation of iron status, and hemoglobin electrophoresis.

The overall ethnicity of patients studied included 57% of Caucasian (including 53.4% Italian), 27.1% African, 10.1% Asian, and 5.5% Hispanic.

A total of 1789 subjects were identified with genetic variants in the globin genes, of which 966 (53.9%) had variations in the β-gene, 478 (26.7%) had variations in the α-gene, and 307 (17.1%) had variations in both α- and β-genes.

A total of 966 patients were carriers of β-globin mutations (Table 1). Of these, 82.9% (802/966) were heterozygous, 6.7% (65/966) homozygous, and 10.2% (99/966) were compound heterozygotes for two different variants. Codon 39 was the most frequent mutation (241/966; 24.9%), identified in heterozygosis in 23.2% (225/966) of patients and in homozygosis in 1.7% (16/966). HbS was identified in 15.9% of cases (155/966); of these, 13% (127/966) of the patients analyzed were heterozygous and 2.9% (28/966) were homozygous. Among the other β-globin variants, the IVSI-110G>A was identified in heterozygosity in 6.3% (61/966) of patients; IVSI-1 G>A was detected in 5% (48/966), and IVSI-6T>C (35/966) in 3.6% of the subjects analyzed. Overall, a total of 847 different β-gene mutations were identified, of which 272 were variants expected to lead to partial loss of β-globin function.

Among the 480 subjects with α-thalassemia variants (Table 2), the α-3.7 variant was the most common (274/480) corresponding to 57% of the subjects carrying α-thalassemia. Of these, 74 subjects (27%) were homozygous for the −3.7 deletion and three of them also had the Hb G Philadelphia variant.

Individuals with heterozygous α-3.7/αα genotype represent 54.7% (150/274), of the subjects carrying the α-3.7 variant. However, several combinations of heterozygous α-3.7 genotype with other variants were also identified including Hb G Philadelphia (9/274, corresponding to 3.3%), IVSI-5nt (6/274) 2.2%, Hb Hasharon (5/274, of Italian origin) 1.8%, α-4.2 (5/274) 1.8%, -- MED (4/274) 1.4%, --SEA (4/274) 1.4%, Initiation codon T-C (3/274) 1.1%, Poly-A c.*93_*94delAA (5/274) 0.7%.

Additionally, we identified 40 subjects carrying unique genotypes including either a single (25 subjects) or multiple heterozygous variants (15 subjects) in the α-globin cluster (Supplementary Table S1). Of the 15 subjects with multiple heterozygous variants in the α globin cluster, 10 were compound heterozygotes for the −3.7. Of these, 5 subjects had additional variants in HbA2, 3 subjects in HBA1 while triple α and –FIL variants were detected in one subject each.

Three hundred and ten individuals were composite carriers of both α- and β-globin variations (Table 3). Among composite α- and β-thalassemia carriers 25.8% (80/310) of genotypes consisted of common deletions α-3.7 in addition to hemoglobin variants HBB:c.20A>T (HbS). The composite ααα/HBB:c.118C>T (Codon39 C>T) and α-3.7/HBB:c.19G>A (HbC) were the second and the third most frequent genotypes, being present in 8.38% (26/310) and 6.77% (21/310) of the cases, respectively. Several rare α-hemoglobin variants were simultaneously identified with β-classical variations: HBA2:c.113C>T (Hb Manawatu)/α–3.7/HBB:c.20A>T (HbS); HBA2:c.358C>G (Hb Lakeview Terrace)/HBB:c.79G>A (HbE); HBA1:c.154G>A (Hb Riccarton)/HBB:c.92+1G>A (IVSI-1). Among composite α- and β-thalassemia, two subjects were found to harbor further additional variants including δ-gene mutations HBB:c.364G>C (Hb D-Los Angeles)/α–4,2/HBD:c.-118 C>T, and HBB:c.93-21G>A (IVSI-110G>A)/ααα/HBD:c.315+1G>A and two with γ-mutations HBB:c.118C>T (Codon39 C>T) omo/– 196 C>T nd HPFH/α-3.7 and HBB:c.315+1G>A (IVSII-1 G>A)/HBG1:c.-211C>T (Cretan HPFH) homozygous.

Additionally, we observed 36 subjects (2.0%) with variations in the delta gene and 11 (0.6%) with variations in the γ-gene (Table 4).

## 4. Discussion

The advent of next-generation sequencing (NGS) has revolutionized diagnostic processes in the molecular analysis of α- and β-globin genes [20,21,22], allowing for the detection of a wider range of genetic variations as well as a more detailed, accurate, and high-throughput analysis.

Our evaluation of the NGS targeting strategy highlighted its ability to enhance the molecular analysis of α- and β-globin genes, offering more comprehensive and accurate diagnostic capabilities. Using the NGS Devyser Thalassemia kit, we studied 2649 subjects and confirmed the diagnosis of α- and/or β-thalassemia in 1789 patients. This approach demonstrated a high diagnostic sensitivity of about 99.9%, providing detailed insights into the genetic basis of the condition and demonstrating the method’s effectiveness in detecting genetic variations related to thalassemia. Another relevant aspect of this technology is the possibility of detecting simultaneously in a single experiment the presence of point mutations, small or large deletions/duplications involving a single gene, or larger anomalies encompassing both the α- or β-loci, allowing significant time and labor savings.

The ability to analyze both point mutations and large deletions simultaneously was very useful in the case of a patient who came to our attention with a suspicion of hereditary persistence of fetal hemoglobin (HPFH) as he had 26% HbF. This suspicion would have led us to first exclude a deletion in the β-globin locus using MLPA analysis. Instead, NGS analysis identified a point mutation in the HBA1 gene [HBA1:c.344C>G p.(Pro115Arg)] producing an abnormal hemoglobin, Hb Chiapas, which co-elutes in the HbF window. Indeed, the patient showed a silent phenotype.

The use of the NGS kit facilitated the identification of variants in both the α- and β-globin genes in 307 patients, with significant practical implications. This analysis is particularly useful for those subjects carrying silent hemoglobin mutations or variants, such as Hb Camperdown or Hb O Arab, and for identifying unstable hemoglobins that escape the first hematological level of analysis and whose phenotypic consequences are often unknown when combined with other α- and β-hemoglobin variants.

Some new variants of uncertain significance, such as single or double heterozygous variants c.-80C>A, c.-138C>T, c.-136C>A in the HBB gene, are difficult to interpret. Their classification represents a challenge in clinical genetics because their impact on protein function and disease phenotype is not immediately clear. In these cases, familial segregation studies, international databases, bioinformatics tools, and population studies have been helpful. However, it has not always been possible to predict the expected phenotype.

The NGS kit provides a more complete understanding of the genetic profile of patients in whom CNVs and SNVs are suspected at the same time and therefore require complementary techniques such as Sanger sequencing and MLPA. However, in one case the NGS analysis provided a false negative result, since the patient carried both the −3.7 deletion and the triple anti-α 3.7 in the α-globin locus, highlighting that this technique based on amplicon enrichment does not allow the evaluation of balanced CNVs.

This kit, tailored for analyzing the α- and β-globin genes, does not completely cover the entire coding regions of the delta- and γ-globin genes. However, it can detect variations in the intronic and proximal coding regions and can still provide useful information on these less commonly analyzed genes. In the retrospective analysis of our patients, we were able to identify solely 18 patients who were also carriers of variants in the delta-globin gene and four patients with variants in the γ-globin genes.

In particular, the NGS kit allowed us to highlight in three patients the co-inheritance of large deletions that removed the adult δ- and β-genes, leading to hereditary persistence of fetal hemoglobin (HPFH) and an α-3.7 kb deletion. This interesting genetic scenario has significant implications for hemoglobin function and clinical phenotype, leading to a milder phenotype. Understanding this interaction is crucial for accurate diagnosis, effective management, providing genetic counseling, and is essential to consider the overall risk assessment of the couples. Furthermore, the high homology of the HBA1, HBA2 genes for the α-globin cluster does not allow a distinction of the specific variants which therefore must be confirmed with the traditional Sanger method.

The Amplicon suite software provides direct access to the HbVar [12], Ithanet [23], Clinvar [24], dbSNP [25], and gnomAD variant database [26], which are crucial resources for variant classification [27,28,29]. Also, it enables the creation of an internal mutation database, which is available and searchable at each subsequent analysis, enhancing the efficiency and accuracy of the diagnostic process. However, the presence of multiple samples with deletions/duplications in the same session sometimes did not allow the algorithm to clearly call the most frequent CNVs such as α-3.7 deletions, α-4.2 deletions and anti-3.07 duplications, which represent 27%, 4.6%, and 0.9% of the analyzed cohort, respectively and therefore required validation by MLPA analysis.

## 5. Conclusions

NGS analysis conducted in a total cohort of 2649 individuals, recruited in four Italian medical genetics laboratories, led to the identification of a wide range of point mutations, deletions and variants in the α- and β-globin genes and in the promoters of the delta- and γ-globin genes. This comprehensive approach, particularly in a multi-ethnic context, such as the one prevalent in Italy, significantly reduced analysis times and allowed the specific characterization of population-specific mutations. It also facilitated the identification of silent hemoglobin variants in genes not directly related to the indication for testing, thus contributing to a better genotypic characterization of the patient. However, NGS also revealed variants with unclear clinical significance, which required further investigation and family segregation studies to determine the possible phenotypic impact.

## Figures and Tables

**Table 1:** β-gene variations. (* Heterozygous; ** Homozygous,*** Compound Heterozygous).

HBB			
HGVS	Protein	Mutation	N° of Samples
NM_000518.5:c.[118C>T];[=]	p.(Gln40 *)	cd39C>T *	223
NM_000518.5:c.[20A>T];[=]	p.(Glu7Val)	HbS *	127
NM_000518.5:c.[93−21G>A];[=]	p.?	IVSI-110G>A *	65
NM_000518.5:c.[92+1G>A];[=]	p.?	IVSI-1G>A *	49
NM_000518.5:c.[19G>A];[=]	p.(Glu7Lys)	HBC *	37
NM_000518.5:c.[79G>A];[=]	p.(Glu27Lys)	HbE *	35
NM_000518.5:c.[ 92+6T>C];[=]	p.?	IVSI-6T>C *	35
NM_000518.5:c.[20A>T];[20A>T]	p.(Glu7Val); p.(Glu7Val)	HbS **	28
NM_000518.5:c.[−151C>T];[=]	p.?	−101C>T *	27
NG_000007.3:g.64336_77738del13403	p.?	δβ-Sicilian *	25
NM_000518.5:c.[316−106C>G];[=]	p.?	IVSII-745C>G *	24
NM_000518.5:c.[364G>C];[=]	p.(Glu122Gln)	Hb D-Los Angeles *	21
NM_000518.5:c.[93-21G>A];[93-21G>A]	p.?	IVSI-110G>A **	20
NM_000518.5:c.[92+5G>C];[=]	p.?	IVSI-5G>C *	16
NM_000518.5:c.[118C>T];[118C>T]	p.(Gln40 *)	cd39C>T **	16
NM_000518.5:c.[315+1G>A];[=]	p.?	IVSII-1G>A *	10
[NG_000007.3:g.63632_71046del];[=]	p.?	Hb Lepore–Boston–Washington *	10
NM_000518.5:c.[20del];[=]	p.(Glu7Glyfs * 13)	cd6-A *	10
NM_000518.5:c.[19G>A];[ 19G>A]	p.(Glu7Lys)	HbC **	9
NM_000518.5:c.[19G>A];[20A>T]	p.(Glu7Lys);p.(Glu7Val)	HbC; HbS ***	6
NM_000518.5:c.[-138C>T];[=]	p.?	−88C>T *	5
NM_000518.5:c.[79G>A];[364G>C]	p.(Glu27Lys)/p.(Glu122Gln)	HbE; HbD-Los Angeles ***	5
NM_000518.5:c.[-136C>A];[=]	p.?	−86C>A *	4
NM_000518.5:c.[25_26del];[=]	p.(Lys9Valfs * 14)	CD8-AA *	4
NM_000518.5:c.[92+6T>C];[92+6T>C]	p.?	IVSI-6T>C **	4
NM_000518.5:c.[27_28insG];[=]	p.(Ser10Valfs * 14)	Codons 8/9(+G) *	3
NM_000518.5:c.[93−21G>A];[118C>T]	p.?	IVSI-110 G>A; cd39C>T ***	3
NM_000518.5:c.[20A>T];[92+1G>A]	p.(Glu7Val)	HbS;IVSI-1G>A ***	3
NM_000518.5:c.[67G>C];[=]	p.(Glu23Gln)	Hb D-Iran *	3
NM_000518.5:c.[126_129del];[=]	p.(Phe42Leufs * 19)	CD41/42-TTCT *	3
NM_000518.5:c.[135del];[=]	p.(Phe46Leufs * 16)	cd44-C *	3
NM_000518.5:c.[364G>A];[=]	p.(Glu122Lys)	Hb-O-Arab *	3
NM_000518.5:c.[20A>T];[118T>C]	p.(Glu7Val); p.(Gln40 *)	HbS; cd39C>T ***	2
NM_000518.5:c.[92G>C];[=]	p.(Arg31Thr)	Hb Monroe *	2
NM_000518.5:c.[315G>C];[=]	p.(Arg105Ser)	Hb Camperdown *	2
NM_000518.5:c.[-151C>T];[315+1G>A]	p.?	−101C>T ; IVSII-1G>A ***	2
NM_000518.5:c.[−142C>T];[=]	p.?	−92C>T *	2
NM_000518.5:c.[−137C>G];[118C>T]	p.?	−87C>G;cd39 ***	2
NM_000518.5:c.[79G>A];[164_168delins GGCATCA]	p.(Glu27Lys)/p.(Val55Glyfs * 8)	HbE; - ***	2
NM_000518.5:c.[20A>T];[316−106C>G]	p.(Glu7Val);p.?	HbS; IVSII-745C>G ***	2
NM_000518.5:c.[23_26dup];[=]	p.(Ser10Glufs * 15)	cd8/9(+AGAA) *	2
NM_000518.5:c.[92+5G>C];[79G>A]	p.?/p.(Glu27Lys)	IVSI-5G>C;HbE ***	2
NM_000518.5:c.[143_144insA];[=]	p.(Asp48Glufs * 6)	Codon 47(+A) *	2
NM_000518.5:c.[155del];[=]	p.(Pro52fs)	cd51(−C) *	2
NM_000518.5:c.[157G>A];[=]	p.(Asp53Asn)	Hb Osu Christiansborg *	2
NM_000518.5:c.[184A>T];[=]	p.(Lys62 *)	cd61 A>T *	2
NM_000518.5:c.[316−1del];[=]	p.?	IVSII-850–G *	2
NM_000518.5:c.[-151C>T];[93-21G>A]	p.?	−101C>T; IVSI-110G>A ***	2
NM_000518.5:c.[79A>G];[20A>T]	p.(Glu27Lys);p.(Glu7Val)	HbE;HbS ***	2
NM_000518.5:c.[20A>T];[316-70C>G]	p.(Glu7Val);p.?	HbS;IVSII-781C>G ***	2
NM_000518.5:c.[22_24del];[=]	p.(Glu8del)	Hb Leiden *	2
NM_000518.5:c.[93−21G>A];[20A>T]	?;p.(Glu7Val)	IVSI-110G>A;HbS ***	2
NM_000518.5:c.[79G>A];[92+5G>C]	p.(Glu27Lys);p.?	HbE;IVSI-5G>C ***	2
NM_000518.5:c.[93−21G>A];[NG_000007.3:g.64336_77738del13403]	p.?	IVSI-110G>A; δβ-Sicilian ***	2
NM_000518.5:c.[118C>T];[NG_000007.3:g.64336_77738del13403]	p.(Gln40 *)/p.?	Cd39;δβ-Sicilian ***	2

**Table 2:** α-gene variations. (* Heterozygous; ** Homozygous,*** Compound Heterozygous).

HBA1-HBA2			
HGVS	Protein	Mutation	N° of Samples
NG_000006.1:g.[34247_38050del];[=]	p.?	α–3.7 *	150
NG_000006.1:g.[34247_38050del];[34247_38050del]	p.?;p.?	α–3.7;α–3.7 **	71
HBA2:c.[95+2_95+6del];[=]	p.?	IVSI-1 (−5nt) *	31
NG_000006.1:g.[34247_38050dup];[=]	p.?	αanti3.7 *	29
HBA2:c.[2T>C];[=]	p. (Met1?)	Init CD ATG>ACG [Met>Thr] *	13
HBA2:c.[207C>G];[α–3.7]	p.(Asn69Lys);p.?	Hb G Philadelphia; α–3.7 ***	9
NC_000016.10:g.[169818_174075del];[=]	p.?	α–4.2 *	7
HBA2:c.[95+2_95+6del];[ 95+2_95+6del]	p.?;p.?	IVSI-1 (-5nt); IVSI-1 (-5nt) **	7
NG_000006.1:g.15164_37864del22701	p.?	α-20.5 *	6
HBA2:c.[95+2_95+6del];[ NG_000006.1:g.[34247_38050del];[34247_38050del	p.?;p.?	IVSI-1(-5nt); α–3.7 ***	6
NG_000006.1:g.[(23641_23662)_(41183_41203)del];[=]	p.?	––MED [TIPO I] *	6
NC_000016.10:g.[151641_182316del];[=]	p.?	––FIL *	6
NC_000016.10:g.[165401_184701del];[=]	p.?	––SEA *	6
HBA1:c. [95+38C>T];[=]	p.?	IVSI-38 C>T *	5
HBA2:c.[142C>G];[=]	p.(Asp48His)	(Hb L-Ferrara, Hb Michigan-I, Hb Michigan-II, Hb Sealy, Hb Sinai) *	5
HBA2:c.[*93_*94del];[=]	p.?	Poly A (AATAAA>AATA--) *	5
NC_000016.10:g.[169818_174075del];[ NG_000006.1:g.[34247_38050del	p.?;p.?	α–4.2/α–3.7 ***	5
HS40	p.?	/ *	5
HBA1: c.[325del];[=]	p.(Thr109Profs * 25)	/ *	4
HBA1:c.[47G>A];[=]	p.(Gly16Asp)	Hb I-Interlaken *	4
NG_000006.1:g.[(23641_23662)_(41183_41203)del];[34247_38050del]	p.?;p.?	--MED [TIPO1]; α–3.7 ***	4
NC_000016.10:g.[165401_184701del]; NG_000006.1:g.[34247_38050del]	p.?;p.?	––SEA;α–3.7 **	4
HBA2:c.[2T>C]; NG_000006.1:g.[34247_38050del]	p. (Met1?);p.?	Init CD ATG>ACG [Met>Thr]; α–3.7 ***	3
HBA2:c.[96-1G>A];[=]	p.?	IVSI-117G>A *	3
HBA1:c.[79G>A]; HBA2:c.[391G>C]	p.(Ala27Thr); p.(Ala131Pro)	Hb Caserta; Hb Sun Prairie [Hb Southern Italy] ***	3
HBA2:c.207C>G];NG_000006.1:g.[34247_38050del]	p.(Asn69Lys);p.?	Hb G Philadelphia; α–3.7 hom ***	3
HBA1:[c.226G>A];[=]	p.(Asp76Asn)	[Hb Matsue-Oki] *	3
HBA1:c.[328delC];[=]	p.(Leu110Trpfs * 24)	α1 Codon 108 –C *	3
HBA2:c.[410T>C];[=]	p.(Leu137Pro)	Hb Bibba *	3
NG_000006.1:g.[34247_38050dup];[34247_38050dup]	p.?	αααanti3,7/αααanti3,7 **	3
NG_000006.1:g.[(7740_9712)_(39907_41156)del];[=]	p.?	––MED [TIPO2] *	3
NG_000006.1:g.[32867_38062del5196];[=]	p.?	-(α)5.2 *	3
HBA1:c.[358C>T];[=]	p.(Pro120Ser)	Hb Groene Hart *	2
HBA2:c.[2T>C];[95+2_95+6del]	p. (Met1?);p.?	Init CD ATG>ACG [Met>Thr]/IVSI-1 -5nt ***	2
HBA2:c.[161C>A];[=]	p.(Ala54asp)	HbJ Rovigo *	2
HBA2:c.[223G>A];[=]	p.(Asp75Asn)	HbG-Pest *	2
HBA2:c.[373T>C];[=]	p.(Ser124pro)	Hb Policoro *	2
HBA2:c.[427T>A];[=]	p.(* 143Kext * 31)	Hb Icaria *	2
HBA2:c.[*93_*94del]; NG_000006.1:g.[34247_38050del]	p.?;p.?	/ ***	2
HBA2:c.[*94A>G];[=]	p.?	Poly A (AATAAA>AATAAG) (αPolyA1, αT-Saudi) *	2
HBA2:c.[2T>C];NG_000006.1:g.(18148_18200)_(37868_37901)del]	p.(Met1?); p.?	Init CD ATG>ACG [Met>Thr]; -20.5 ***	2

**Table 3:** Composite α- and β thalassemia variants.

HGVS	Protein	Mutations	N°of Samples
[HBB:c.20A>T];[α-3.7]	p.(Glu7Val);p.?	HbS/α-3.7	81
[HBB:c.118C>T];[αααanti3.7]	p.(Gln40 *);p.?	CD39 C>T/αααanti3.7	26
[HBB:c.19G>A];[α-3.7]	p.(Glu7Lys)/p.?	HbC/α-3.7	21
[HBB:c.20A>T];[ α-3.7/α-3.7]	p.(Glu7Val)/p.?	HbS/α3.7α-3.7	20
[HBB:c.20A>T;20A>T];[α-3.7]	p.(Glu7Val)/p.?	HbS/α-3.7	13
[HBB:c.118C>T];[α-3.7]	p.(Gln40 *)/p.?	cd39/α-3.7	11
[HBB:c.20A>T ;20A>T];[α-3.7;α-3.7]	p.(Glu7Val)/p.?	HbS;HbS/α-3.7α-3.7	6
[HBB:c.20A>T];[HBA2:c.207C>G(;)α–3.7]	p.(Glu7Val);p.(Asn69Lys)/p.?	HbS/Hb G Philadelphia/α-3.7	6
[HBB:c.19G>A];[α-3.7/α-3.7]	p.(Glu7Val);p.?	HbC/α-3.7α−3.7	4
[HBB:c.118C>T];[HBD:c.14C>T]	p.(Gln40 *); p.(Thr5Ile)	CD39/Codon 4	4
[HBB:c.92+1G>A];[αααanti3.7]	p.?;p.?	IVSI-1/αααanti3.7	3
[HBB:c.93-21G>A];[αααanti3.7]	p.?;p.?	IVSI-110G>A/αααanti3.7	3
[HBB:c.364G>C];[α-3.7]	p.(Glu122Gln);p.?	Hb D-Los Angeles/α-3.7	3
[HBB:c.20A>T];[HBA2:c.75T>G(;)α-3.7]	p.(Glu7Val) ;p.(Tyr25Ter)/p.?	HbS/CD24T>G/α-3.7	3
[HBB:c.93-21G>A];[α-3.7]	p.?;p.?	IVSI-110G>A/α-3.7	3
[HBB:c.316-106C>G];[αααanti3.7]	p.?;p.?	IVSII-745C>G/αααanti3.7	3
[HBB:c.79G>A];[α-3.7]	p.(Glu27Lys);p.?	HbE/α-3.7	3
[HBAα–3.7];[HBD:c.-118C>T]	p.?;p.?	α-3.7/δ-68 C->T	2
[HBB:c.19G>A;19G>A];[α-3.7]	p.(Glu7Val);p.(Glu7Val);p.?	HbC/HbC/α-3.7	2
[HBB:c.20A>T];[αααanti-3.7]	p.(Glu7Val);p.?	HbS/αααanti3.7	2
[HBB:c.20A>T;92+5G>C];[α-3.7]	p.(Glu7Val)/p.?;p.?	HbS/IVSI-5G>C/α-3.7	2
[HBB:c.92+6T>C];[α–3.7]	p.?;p.?	IVSI-6T>C/α-3.7	2
[HBB:c.93-21G>A];[HBA2:c. * 94A>G]	p.?;p.?	IVSI-110G>A/Poly A (A->G)	2
[NG_000007.3:g.54867_139178del];[α-3.7]	p.?;p.?	HPFH-2 Ghanaian/α-3.7	2
[HBB:c.79G>A];[NG_000006.1:g.26264_45564del]	p.(Glu27Lys);p.?	HbE/--SEA	2

**Table 4:** HBD and HBG variants.

HBD Variants			
GVS	Protein	Mutation	N° of Samples
NM_000519.4:c.[14C>T];[=]	p.(Thr5Ile)	cd4 ACT>ATT	19
NM_000519.4:c.[-118 C>T];[=]	/	−68 (C>T)	10
NG_000007.3:g.57237_64443del7207 (-7.2Kb)	/	–7.2 kb Corfu deletion	4
NM_000519.4:c.[-115A>G];[=]	/	−65 (A>G)	2
**HBG Variants**			
**HGVS**	**Protein**	**Mutation**	**N° of Samples**
NC_000011.10:g.5158438_5242749del	/	HPFH-2 Ghanaian	3
–175 C>T	/	HPFH	2

## Data Availability

The original contributions presented in this study are included in the article and in the Supplementary Materials.

## Supplementary Materials

The following supporting information can be downloaded at: https://www.mdpi.com/article/10.3390/genes16010028/s1, Table S1: Rare variant identified.
