# Supplementary material for: Relevance of Next-Generation Sequencing in the Diagnosis of Thalassemia and Hemoglobinopathies: The Experience of Four Italian Diagnostic Hubs
Source: Genes (Basel). 2024 Dec 27;16(1):28. doi: 10.3390/genes16010028 (PMC11765070; doi:10.3390/genes16010028)
Supplement: Supplementary file 1 [file genes-16-00028-s001.zip › genes-3372072-supplementary.pdf]

Tab.S1 Rare variant identified			
**HGVS**	**Protein**	**Mutation common name**	**n°**
HBB:c.[-140C>T];[79G>A]	p.?; p.(Glu27Lys)	-90C>T; HbE	1
HBB:c.[341T>A];[=]	p.(Val114Glu)	Hb New York	1
HBB:c.[315+1G>A];[315+1G>A]	p.?	IVSII-1G>A	1
HBB:c.[394C>G];[=]	p.(Gln132Glu)	Hb Camden	1
HBB:c.[*96T>C];[=]	p.?	+1570 C>T	1
HBB:c.[*96T>C];[118C>T]	p.?; p.(Gln40*)	+1570 C>T; CD39 C>T	1
HBB:c.[-151C>T];[20A>T]	p.?; p.(Glu7Val)	-101C->T;HbS	1
HBB:c.[-151C>T];[-151C>T]	p.?	-101C->T;-101C->T	1
HBB:c.[-138C>T];[-138C>T]	p.?	-88C>T	1
HBB:c.[-138C>T];[c.19G>A]	p.(Glu7Lys)	-88C>T; HBC	1
HBB:c.[-137C>G];[=]	p.?	-87C>G	1
HBB:c.[-137C>G];[-137C>G]	p.?	-87C>G	1
HBB:c.[-106G>C];[=]	p.?	-56G>C	1
HBB:c.[-79A>G];[=]	p.?	-29A>G	1
HBB:c.[8A>C];[=]	p.(His3Pro)	Hb Agrigente	1
HBB:c.[19G>A];[404T>C]	p.(Glu7Lys);p.(Val135Ala)	HBC; Hb Yaounde	1
HBB:c.[20A>T];[3G>A]	p.(Glu7Val); p.(Met1?)	HbS; init CDATG>ATA	1
HBB:c.[20A>T];[92+6T>C]	p.(Glu7Val);p.?	HbS; IVSI-6T>C	1
HBB:c.[20del];[-240G>A]	p.(Glu7Glyfs*13);p.?	cd6-A;-190 G>A	1
HBB:c.[20A>T];[113G>A]	p.(Glu7Val); p.(Trp38*)	HbS;CD37G>A	1
HBB:c.[20A>T];[364G>A]	p.(Glu7Val); p.(Glu122Lys)	HbS; Hb-O-Arab	1
HBB:c.[20A>T];[NG_000007.3:g.64336_77738del13403]	p.(Glu7Val); p.?	HbS; Sicilian (deltabeta)	1
HBB:c.[23A>G];[=]	p.(Glu8Gly)	Hb G-San José	1
HBB:c.[23A>G];[c.93-21G>A]	p.(Glu8Gly);p.?	Hb G-San José; IVSI-110G>A	1
HBB:c.[27_28insG];[51delC;c.33C>A]	p.(Ser10Valfs*14);p.(Lys18Argfs*2);p.(Ala11=)	CD 8/9; CD 16	1
HBB:c.[46delT];[=]	p.(Trp16fs*4)	CD15 -T	1
HBB:c.[50G>A];[=]	p.(Gly17Asp)	Hb J Baltimore	1
HBB:c.[75T>A];[=]	p.(Gly25=)	CD 24T>A	1
HBB:c.[82G>T];[=]	p.(Ala28Ser)	Hb Knossos	1
HBB:c.[82G>T];[316-70C>G]	p.(Ala28Ser);p.?	Hb Knossos; IVS2-781	1
HBB:c.[92G>C];[-92C>G]	p.(Arg31Thr);p.?	Hb Monroe; -42 C>G	1
HBB:c.[92+1G>A];[93-21G>A]	p.?	IVSI-1; IVSI-110 G>A	1
HBB:c.[92+6T>C];[*111A>G]	p.?	IVSI-6 T>C; Poly A (A->G)	1
HBB:c.[92+6T>C];[*112A>G]	p.?	IVSI-6 T>C; Poly A (A->G)	1
HBB:c.[92+2T>A];[=]	p.?	IVSI-2 T>A	1
HBB:c.[92+5G>C];[c.92+5G>C]	p.?	IVSI-5G>C; IVSI-5G>C	1

**Tab.S1 Rare variant identified**			
**HGVS**	**Protein**	**Mutation common name**	**n°**
HBB:c.[93-21G>A];[135delC]	p.?; p.(Phe46Leufs*16);	IVSI-110 G>A;CD44 -C	1
HBB:c.[93-21G>A];[-137C>G]]	p.(Phe46Leufs*16);p.?	IVSI-110 G>A;-87 C>G	1
HBB:c.[93-1 G>A];[=]	p.?	IVSI-130G>A]	1
HBB:c.[103G>T];[=]	p.(Val35Phe)	Hb Pitie Sal Petriere	1
HBB:c.[113G>A];[=]	p.(Trp38*)	CD37G>A	1
HBB:c.[118C>T];[19G>A]	p.(Gln40*); p.(Glu7Lys)	CD39 C>T;HBC	1
HBB:c.[118C>T];[-106G>C]	p.(Gln40*);p.?	CD39 C>T;-56G>C	1
HBB:c.[118C>T];[135delC]	p.(Gln40*); p.(Phe46fs*16)	CD39 C>T; CD44-C>A	1
HBB:c.[118C>T];[376C>A]	p.(Gln40*); p.(Pro126Thr)	CD39 C>T; Hb Novara	1
HBB:c.[155delC];[*96T>C]	p.(Pro52fs*10);p.?	CD51 –C; +1570 C>T	1
HBB:c.[155C>G];[=]	p.(Pro52Arg)	Hb Willamette	1
HBB:c.[208G>A];[=]	p.(Gly70Ser)	Hb City of Hope	1
HBB:c.[230C>A];[230C>A]	p.(Ala77Asp); p.(Ala77Asp)	Hb J-Chicago (CD76)	1
HBB:c.[230C>A];[=]	p.(Ala77Asp)	Hb J-Chicago (CD76)	1
HBB:c.[262A>C];[=]	p.(Thr88Pro)	Hb Valletta	1
HBB:c.[269G>A];[=]	p.(Ser90Asn)	Hb Creteil	1
HBB:c.[271G>A];[=]	p.(Glu91Lys)	Hb Agenogi	1
HBB:c.[280T>C];[=]	p.(Cys94Arg)	Hb Okazaki	1
HBB:c.[298G>A];[=]	p.(Asp100Asn)	Hb Kempsey	1
HBB:c.[314G>A];[=]	p.(Arg105Lys)	Hb Alzette	1
HBB:c.[316-70C>G];[=]	p.?	IVSII-781	1
HBB:c.[316-146T>G];[*96T>C]	p.?	IVSII-705T>G;+1570 C>T	1
HBB:c.[316-106C>G];[364G>A] []	p.?; p.(Glu122Lys)	IVSII-745; Hb-O-Arab	1
HBB:c.[316-197C>T];[=]	p.?	IVSII-654C>T	1
HBB:c.[316-2A>C];[=]	p.?	IVSII-849A>C	1
HBB:c.[316-3C>A];[=]	p.?	IVSII-848C>A	1
HBB:c.[315+4_315+5delAG];[=]	p.?	IVSII-4,5 (-AG)]	1
HBB:c.[*111A>G];[=]	p.?	PolyA (A>G)	1
HBB:c.[338G>T];[=]	p.(Cys113Phe)	Hb Canterbury	1
HBB:c.[364G>A];[364G>A]	p.(Glu122Lys);p.(Glu122Lys)	Hb-O-Arab	1
HBB:c.[371C>A];[=]	p.(Thr124Asn)	Hb Ernz	1
HBB:c.[380T>A];[=]	p.(Val127Glu)	Hb Hofu	1
HBB:c.[380T>G];[=]	p.(Val127Gly)	Hb Dhonburi	1
HBB:c.[386C>A];[=]	p.(Ala129Asp)	Hb J-Guantanamo	1
HBB:c.[395A>G];[=]	p.(Gln132Arg)	Hb Sarrebourg	1
HBB:c.[397A>C];[=]	p.(Lys133Gln)	Hb K Woolwich	1
HBB:c.[407C>T];[=]	p.(Ala136Val)	Hb Alperton	1

Tab.S1 Rare variant identified

HGVS	Protein	Mutation common name	n°
HBB:c.[410G>A];[=]	p.(Gly137Asp	Hb Hope	1
NG_000007.3:g.[52524_60162del;66278_73952del];[=]	p.?	Turkish 7.6 kb deletion	1
NG_000007.3:g.[63632_71046del];[g.63632_71046del]	p.?	Hb Lepore-Boston-Washington	1
HBB:c.[*108_*112delAATAA];[=]	p.?	Poly A (-AATAA)	1
HBB:c.[380T>C];[=]	p.(Val127Gly)	Hb Beirut	1
HBB:c.[*96T>C];[-142C>T]	p.?	+1570 C>T;-92C>T	1
HBA2:c.[2T>C];[--FIL]	p.(Met1?)	Init CD T>C; --FIL	1
HBA1:c.[-3_-2delAC];[α-3.7]	p.?	α-3.7	1
HBA1:c.[29A>G];[=]	p.(Asn10Ser)	Hb Anadour	1
HBA2:c.[38C>A];[=]	p.(Ala13Asp)	Hb J-Paris-I	1
HBA2:c.[43T>C];[=]	p.(Trp15Arg)	Hb Evanston	1
HBA2:c.[45G>C];[α-3.7]	p.(Trp15Cys)	Hb Bladensburg;α-3.7	1
HBA1:c.[63C>A];[=]	p.(His21Gln)	Hb Brugg	1
HBA2:c.[79G>A;c.391G>C;c.391G>C];[α-3.7]	p.(Ala27Thr); p.(Ala131Pro);p.?	Hb Caserta; Hb Sun Prairie (=Hb Southern Italy); α-3.7	1
HBA2:c.[79G>A;c.391G>C];[α-3.7]	p.(Ala27Thr); p.(Ala131Pro);p.?	Hb Caserta; Hb Sun Prairie (=Hb Southern Italy); α-3.7	1
HBA2:c.[82G>A];[=]	p.(Glu28Lys)	Hb Shuanfeng	1
HBA1 c.[95G>C];[=]	p.(Arg32Thr)	Hb Mao	1
HBA1:c.[70 G>T] ;[=]	p.(Glu24*)	Cd23 (GAG>TAG)	1
HBA1:c.[116C>T];[=]	p.(Thr39Ile)	Hb Chelsea	1
HBA2:c.[167T>C];[=]	p.(Val56Ala)	Hb Gerland	1
HBA2:c.[173G>A];[=]	p.(Gly58Asp)	HbJ Norfolk	1
HBA1:c.[187delG];[=]	p.(Val63Trpfs*5)	Hb Champaign	1
HBA1:c[271A>G];[=]	p.(Lys91Glu)	Hb Sudbury	1
HBA1:c.[278G>A];[=]	p.(Arg93Gly)	Hb J-Cape Town	1
HBA2:c.[283G>A];[α-3.7]	p.(Asp95Asn);p.?	Hb Titusville; α-3.7	1
HBA1:c.[328delC];[α-3.7]	p.(Leu110Trpfs*24)	Hb Manhattan; α-3.7	1
HBA1:c.[326C>A];[α-3.7]	p.(Thr109Asn)	Hb Rogliano; α-3.7	1
HBA1:c.[338A>G];[338A>G]	p.(His113Arg); p.(His113Arg)	Hb Serbia	1
HBA2:c.[343C>G];[=]	p.(Pro115Ala)	Hb Broomhill	1
HBA1:c.[344C>G];[=]	p.(Pro115Arg)	Hb Chiapas	1
HBA2 c.[353T>C];[=]	p.(Phe118Ser)	CD117T>C	1
HBA1:c.[358C>T];[α–4.2]	p.(Pro120Ser);p.?	Hb Groene Hart; α–4.2	1
HBA1:c.[377T>C];[=]	p.(Leu126Pro)	Hb Quong Sze	1
HBA1:c.[381C>G];[=]	p.(Asp127Glu)	Hb Burlington	1
HBA2:c.[388delC];[=]	p.(Leu130TrpfsTer4)	Hb Hamilton Hill	1
HBA2:c.[424C>G];[=]	p.(Arg142Gly)	Hb-J-Camaguay	1
HBA2:c.[427T>A];[427T>A]	p.(Ter143Lysext*31); p.(Ter143Lysext*31)	Hb Icaria	1

Tab.S1 Rare variant identified

HGVS	Protein	Mutation common name	n°
HBA2:c.[427T>G];[=]	p.(Ter143Glu)	Hb Seal Rock	1
HBA2:c.[427T>C];[427T>C]	p.(Ter143Glnext*31); p.(Ter143Glnext*31)	Hb Constant Spring (HbCS)]	1
α-3.7/--FIL	p.?	α-3.7/--FIL	1
α-3.7/c.29A>G	p.?; p.(Asn10Ser)	α-3.7; Hb Zurich-Albisrieden	1
α–4.2/ HBA2:c.344C>T [Hb Nouakchott]	p.?; p.(Pro115Leu)	α–4.2;Hb Nouakchott	1
HBA2:c.[41_46del];[=]	p.(Ala14_Trp15del)	Hb Souli	1
HBA2:c.[64G>C];[=]	p.(Ala22Pro)	Hb Fointainbleau	1
ααα$^{anti3.7}$/α-3.7	p.?	ααα$^{anti3.7}$/α-3.7	1
rsa[GRCh38] 16p13.3(37150_171180x1,196320x2)	p.?	/	1
[HBB:c.118C>T];[HBA2:c.2T>C]	p.(Gln40*); p. (Met1?)	CD39 C>T	1
[HBB:c.79G>A];[αααanti3.7]	p.(Glu27Lys);p.?	HbE; αααanti3.7	1
[HBB:c.20A>T];[NG_000006.1:g.32867_38062del5196]	p.(Glu7Val);p.?	HbS; α–5,2	1
[HBB:c.19G>A;20A>T];[α-3.7]	p.(Glu7Lys); p.(Glu7Val)	HBC; HbS; α-3.7	1
[HBB:c.20A>T;20A>T];[αααanti3.7]	p.(Glu7Val); p.(Glu7Val)	HbS; αααanti3.7	1
[HBB:c.118C>T];[HBA2:c.142G>C][α-3.7]	p.(Gln40*); p.(Asp48His)	CD39 C>T; Hb Hasharon; α-3.7	1
[HBB:c.220G>A];[α-3.7;α-3.7]	p.(Asp74Asn)	Hb G-Accra; α-3.7	1
[NG_000007.3:g.63632_71046del];[αααanti3.7]	p.?	Hb Lepore-Boston-Washington/ αααanti3.7	1
[HBB:c.-138C>T;20A>T];[α-3.7]	p.?; p.(Glu7Val)	-88C>T; HbS; α-3.7	1
[HBB:c.92G>C;92G>C];[α-3.7]	p.(Arg31Thr); p.(Arg31Thr)	Hb Monroe	1
[HBB:c.-79A>G];[α-3.7;α-3.7]	p.?	–29 A>G; α-3.7	1
[HBB:c.-136C>A];[α-3.7]	p.?	-86 C>A; α-3.7	1
[HBB:c.19G>A];[α–4.2]	p.(Glu7Lys);p.?	HBC; α–4.2	1
[HBB:c.19G>A;20A>T];[HBA1: c.184_186del]	p.(Glu7Lys); p.(Glu7Val); p.(Lys62del)	HBC; HbS; HbClinic	1
[HBB:c.19G>A];[αααanti3.7]	p.(Glu7Lys);p.?	HBC; αααanti3.7	1
[HBB:c.19G>A;20A>T];[α-3.7;α-3.7]	p.(Glu7Lys); p.(Glu7Val)	HBC; HbS; α-3.7	1
[HBB:c.19G>A];[HBD:c.315+1G>A]*	p.(Glu7Lys);p.?	HBC; IVSII-1G>A	1
[HBB:c.20A>T;93-21G>A];[αααanti3.7]	p.(Glu7Val);p.?	HbS; IVSI-110G>A; αααanti3.7	1
[HBB:c.20A>T];[HBA2:c.113C>T;α-3.7]	p.(Glu7Val)/ p.(Pro38Leu); p.?	HbS;Hb Manawatu;α-3.7	1
[HBB:c.20A>T];[HBD:c.-118C>T]	p.(Glu7Val)/p.?	HbS; -68 (C>T)	1
[HBB:c.20del];[αααanti3.7]	p.(Glu7Glyfs*13);p.?	cd6-A; ααα	1
[HBB:c.20A>T];[HBA2:c.207C>G(;)α-3.7 hom]	p.(Glu7Val); HBA2:p.(Asn69Lys);p.?	HbS; Hb G Philadelphia; α-3.7	1
[HBB:c.20A>T;c.316-70C>G];[α-3.7]	p.(Glu7Val);p.?	HbS; IVS2-781; α-3.7	1
[HBB:c.20A>T;c.-79A>G];[α-3.7]	p.(Glu7Val);p.?	HbS; -29 (A->G); α-3.7	1
[HBB:c.23_26dup];[α-3.7]	p.(Ser10Glufs*15);p.?	CD8/9 +G; α-3.7	1
[HBB:c.25_26del];[α-3.7]	p.(Lys9Valfs*14);p.?	CD8 –AA; α-3.7	1
[HBB:c.23A>G];[α-3.7]	p.(Glu8Gly);p.?	Hb G-San José; α-3.7	1
[HBB:c.79G>A];[HBA2:c.96-1G>A]	p.(Glu27Lys);p.?	HbE; IVSI-117G>A	1

Tab.S1 Rare variant identified

HGVS	Protein	Mutation common name	n°
[HBB:c.79G>A];[HBA2:c.358C>G]	p.(Glu27Lys); p.(Pro120Ala)	HbE; Hb Lakeview Terrace	1
[HBB:c.79G>A];[HBA2:c.427T>C]	p.(Glu27Lys); p.(Ter143Glnext*31)	HbE; Hb Constant Spring (HbCS)	1
[HBB:c.92+1G>A];[HBA1:c.154G>A]	p.?; p.(Gly52Ser)	IVSI-1; Hb Riccarton	1
[HBB:c.92+1G>A];[HBA2:c.95+2_95+6del;α-3.7]	p.?	IVSI-1; IVSI -5nt	1
[HBB:c.92+1G>A];[α-3.7]	p.?	IVSI-1;α-3.7	1
[HBB:c.92+1G>A];[HS40 del]	p.?	IVSI-1; HS40 del	1
[HBB:c.92+5G>C];[ααα$^{anti3.7}$];[HBD:c.-118C>T]	p.?	IVSI-5G>C; ααα$^{anti3.7}$;-68G>T	1
[HBB:c.93-21G>A];[HBA2:c.95+2_95+6del]	p.?;	IVSI-110G>A; IVSI -5nt	1
[HBB:c.20A>T];[HBA2:c.345delC]	p.(Glu7Val);p.(Ala116Profs*18)	HbS;CD114-C	1
[HBB:c.20A>T];[α-3.7;α-20,5]	p.(Glu7Val)	HbS; α-3.7;α-20,5	1
[HBB:c.20A>T;HBB:c.20A>T];[α-3.7; NG_000006.1:g.32867_38062del5196]	p.(Glu7Val);p.(Glu7Val);p.?	HbS; HbS;α-3.7;α -5,2	1
[HBB:c.75T>A];[NG_000007.3:g.48795_127698del78904]	p.(Gly25=);p.?	Codon24(T->A); Chinese (Agammadeltabeta)0-thal)	1
[HBB:c.113G>A];[ααα$^{anti3.7}$]	p.(Trp38Ter);p.?	CD37(TGG>TAG);ααα$^{anti3.7}$	1
[HBB:c.118C>T];[HBA1:c.358C>T]	p.(Gln40*);p.(Pro120Ser)	CD39 C>T; Hb Groene Hart	1
[HBB:c.118C>T]; [ααα^{anti4,2}]	p.(Gln40*);p.?	CD39 C>T; αααanti4,2	1
[HBB:c.118C>T];[α-3.7;α-3.7];[HBD:c.14C>T]	p.(Gln40*);p.?; p.(Thr5Ile)	CD39 C>T;α-3.7;CD4C>T(HbA2-Mitsero)	1
[HBB:c.118C>T];[α-3.7];[HBD:c.14C>T]	p.(Gln40*);p.?; p.(Thr5Ile)	CD39 C>T;α-3.7;CD4C>T(HbA2-Mitsero)	1
[HBB:c.118C>T];[HBD:c.-50A>C]	p.(Gln40*);p.?	CD39 C>T;	1
[HBB:c.118C>T];[α-5,2]	p.(Gln40*);p.?	CD39 C>T;α-5,2	1
[HBB:c.118C>T;HBB:c.118C>T];[HBG1:-249C>T];[α-3.7]	p.(Gln40*);p.(Gln40*);p.?	CD39 C>T;-196C>T the italian nd HPFH	1
[HBB:c.118C>T];[HBA1:c.424C>G]	p.(Gln40*); p.(Arg142Gly)	CD39 C>T; Hb J Camaguay	1
[NG_000007.3:g.64336_77738del13403];[α-3.7]	p.?	db-Sicilian;α-3.7	1
[HBB:c.118C>T];[HBA1:c.392C>T]	p.(Gln40*); p.(Ala131Val)	CD39 C>T; Hb Westborough	1
[HBB:c.262A>C];[ααα$^{anti3.7}$]	p.(Thr88Pro);p.?	Hb Valletta; ααα$^{anti3.7}$	1
[HBD:-118C>T];[ααα$^{anti3.7}$]	p.?	-68G>T;ααα$^{anti3.7}$	1
[HBD:-118C>T;HBD:-118C>T];[ααα$^{anti3.7}$]	p.?	-68G>T;ααα$^{anti3.7}$	1
[HBB:c.364G>C];[ααα$^{anti3.7}$]	p.(Glu122Gln);p.?	Hb D-Los Angeles; ααα$^{anti3.7}$	1
[HBB:c.364G>C];[α-4,2];[HBD:c.-118 C>T]	p.(Glu122Gln);p.?	Hb D-Los Angeles;α-4.2;-68C>T	1
[HBB:c.371C>A];[α-3.7]	p.(Thr124Asn);p.?	Hb Ernz;α-3.7	1
[HBB:c.371C>A];[HBA2:c.*94A>G]	p.(Thr124Asn);p.?	Hb Ernz;	1
[HBB:c.397A>C];[α-3.7]	p.(Lys133Gln);p.?	HB K WOOLWICH/α-3.7	1
[HBB:c.404 T>C];[α-3.7]	p.(Val135Ala);p.?	Hb Yaounde; α-3.7	1
[HBB:c.404 T>C];[α -20.5]	p.(Val135Ala);p.?	Hb Yaounde; α -20.5	1
[NG_000007.3:g.57237_64443del7207;α-3.7]	p.?	–7.2 kb; the Corfu deletion; α-3.7	1
[HBD:c.14C>T];[α-3.7]	p.(Thr5Ile);p.?	CD4C>T (HbA2-Mitsero)	1
[HBD:c.7C>T];[α-3.7]	p.(His3Tyr);p.?	CD2C>T;α-3.7	1
[HBB:c.315+1G>A];[ααα$^{anti3.7}$]	p.?	IVSII-1 G>A; ααα$^{anti3.7}$	1

Tab.S1 Rare variant identified

HGVS	Protein	Mutation common name	n°
[HBB:c.315+1G>A];[HBG1:c.-211C>T;c.-221C>T]	p.?	IVSII-1 G>A; CRETAN HPFH	1
[NG_000007.3:g.71609_72227del619];[HBA1:c.69del]	p.?;p.Glu24Serfs*26	CD22-C	1
[HBB:c.316-106C>G;c.118C>T];[$\alpha\alpha\alpha^{anti3.7}$]	p.(Gln40*)	IVSII-745; CD39 C>T; $\alpha\alpha\alpha^{anti3.7}$	1
[HBB:c.*96T>C];[α-3.7;α-3.7]	p.?	+1570 C>T; α-3.7	1
[HBB:c.316-197C>T;c.-6G>C];[α-3.7]	p.?	IVSII-654 C>T; Hb Odisha; α-3.7	1
[HBB:c.251del];[α-3.7]	p.(Gly84Alafs*6); p.?	Codons 82/83 (-G); α-3.7	1
[HBB:c.374C>A];[α-3.7]	p.(Pro125Gln)	Hb Ty GARD; α-3.7	1
[HBB:c.93-21G>A];[$\alpha\alpha\alpha^{anti3.7}$];[HBD:c.315+1G>A]*	p.?	IVSI-110G>A; $\alpha\alpha\alpha^{anti3.7}$	1
[HBB:c.220G>A;[α-3.7]	p.(Asp74Asn)	Hb G-Accra; α-3.7	1
[NG_000007.3:g.63632_71046del];[HBA1:c.-16T>C]	p.?	Hb Lepore-Boston-Washington	1
[HBB:c.93-21G>A];[α -20.5]	p.?	IVSI-108T>C; α -20.5	1
[HBB:c.*96T>C];[HBA1:c.-59C>T]	p.?	+1570 C>T;	1
HBG1:[c-249C>T]	p.?	-196C>T the italian nd HPFH	1
HBG1:[c-249C>T];[HBG1:c.227T>C]*	p.?	-196C>T the italian nd HPFH; HbF Sardinia	1
[HBB:c.20A>T];[HBD:c.49G>C]*	p.(Glu7Val);p.(Gly17Arg)	HbS;HbA'2	1
[HBB:c.20A>T;c.93-21G>A];[HBD:c.49G>C]*	p.(Glu7Val);p.(Gly17Arg)	HbS; IVSI-110G>A; HbA'2	1
[HBB:c.19G>A];[HBD:c.49G>C]*	p.(Glu7Val);p.(Gly17Arg)	HbC;(HbA'2)	1
HBD:c.[49G>C]*	p.(Gly17Arg)	HbA'2	1
HBD:c.[82G>T]*	p.(Ala28Ser)	Hb A2-Yialousa (CD27)	1
HBD:c.[-118 C>T]	p.?	-68G>T	1
HBD:c.[-115A>G]	p.?	-65A>G	1
HBD:c.[-105T>C]	p.?	-55T>C	1
HBD:c.[316-2A>C]*	p.?	IVSII-897A>C	1
HBD:c.[428C>A] *	p.(Ala143Asp)	HbA2 Fitzroy	1
HBD:c.[301C>T] *	p.(Pro101Ser)	HbA2 Saurashtra	1
HBG1:c.[227T>C]*	p.(Leu76Pro)	Hb F Sardinia	1

*These variants are not identifiable by Devyser kits. But they are identified by Sanger sequencing
